# *N*-(2-Amino­phenyl­sulfonyl)-*N*-(2-nitro­phenyl­sulfonyl)methylamine

**DOI:** 10.1107/S1600536808028092

**Published:** 2008-09-13

**Authors:** Xu-Yun Li, Zu-Wei Song

**Affiliations:** a College of Science, Qingdao Agricultural University, Qingdao 266109, People’s Republic of China

## Abstract

In the title mol­ecule, C_13_H_13_N_3_O_6_S_2_, the two benzene rings form a dihedral angle of 28.59 (7)°. The crystal sructure exhibits weak inter­molecular N—H⋯O, C—H⋯O and C—H⋯N hydrogen bonds and π–π inter­actions [centroid-to-centroid distance = 3.899 (3) Å].

## Related literature

For applications of sulfonimide-containing compounds, see: Kamoshita *et al.* (1987 ▶); Zhang *et al.* (2007 ▶). For the crystal structure of a related compound, see: Henschel *et al.* (1996 ▶).
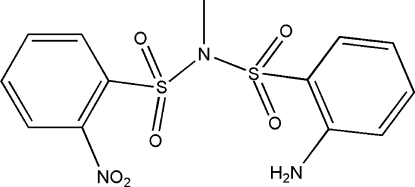


## Experimental

### 

#### Crystal data

C_13_H_13_N_3_O_6_S_2_
*M* *_r_* = 371.38
Orthorhombic, 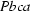

*a* = 13.844 (3) Å
*b* = 12.942 (2) Å
*c* = 16.645 (3) Å
*V* = 2982.2 (10) Å^3^
*Z* = 8
Mo *K*α radiation
μ = 0.40 mm^−1^
*T* = 153 (2) K
0.60 × 0.56 × 0.08 mm

#### Data collection

Rigaku R-AXIS RAPID IP area-detector diffractometer
Absorption correction: multi-scan (*ABSCOR*; Higashi, 1995 ▶) *T*_min_ = 0.733, *T*_max_ = 0.969
34829 measured reflections
2630 independent reflections
2428 reflections with *I* > 2σ(*I*)
*R*_int_ = 0.027

#### Refinement

*R*[*F* ^2^ > 2σ(*F* ^2^)] = 0.032
*wR*(*F* ^2^) = 0.087
*S* = 1.08
2630 reflections
218 parameters
H-atom parameters constrained
Δρ_max_ = 0.42 e Å^−3^
Δρ_min_ = −0.39 e Å^−3^

### 

Data collection: *RAPID-AUTO* (Rigaku, 2004 ▶); cell refinement: *RAPID-AUTO*; data reduction: *RAPID-AUTO*; program(s) used to solve structure: *SHELXTL* (Sheldrick, 2008 ▶); program(s) used to refine structure: *SHELXTL*; molecular graphics: *SHELXTL*; software used to prepare material for publication: *SHELXTL*.

## Supplementary Material

Crystal structure: contains datablocks I, global. DOI: 10.1107/S1600536808028092/cv2436sup1.cif

Structure factors: contains datablocks I. DOI: 10.1107/S1600536808028092/cv2436Isup2.hkl

Additional supplementary materials:  crystallographic information; 3D view; checkCIF report

## Figures and Tables

**Table 1:** Hydrogen-bond geometry (Å, °)

*D*—H⋯*A*	*D*—H	H⋯*A*	*D*⋯*A*	*D*—H⋯*A*
N3—H3*C*⋯O1^ii^	0.88	2.52	3.366 (2)	160
C12—H12*A*⋯O2^ii^	0.95	2.59	3.508 (3)	162
C9—H9*A*⋯N3^iii^	0.95	2.59	3.372 (3)	140

Symmetry codes: (ii) 
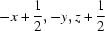
; (iii) 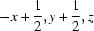.

**Fig. 1.**
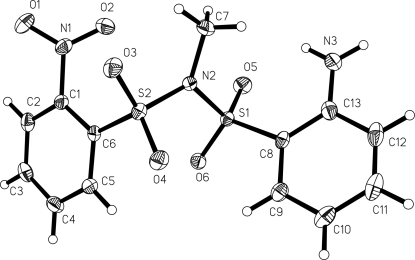
The molecular structure of (I), with atom labels and 40% probability displacement ellipsoids for non-H atoms.

**Fig. 2.**
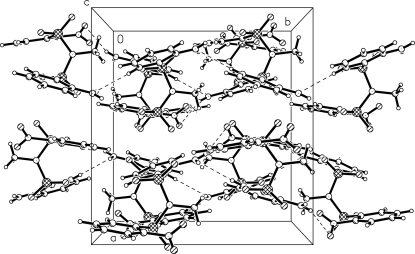
Packing diagram.

## supplementary crystallographic information

### Comment

Many compounds containing sulfonimide groups possess a broad spectrum of biological activities and can be widely used as herbicides (Kamoshita *et al.*,  1987) or catalysts (Zhang *et al.*,  2007). Herein we report the crystal structure of the title compound, (I).

In (I) (Fig. 1), all bond lengths and angles are in a good agreement with those reported previously for related compounds (Henschel *et al.*, 1996). Two benzene rings (C1–C6 and C8–C13) form a dihedral angle of 28.59 (7)°.

The crystal packing exhibits π–π interactions (Table 1) and weak intermolecular N—H···O, C—H···N and C—H···O hydrogen bonds (Table 2).

### Experimental

A solution of 2-aminobenzene-1-sulfonyl chloride (10 mmol) 1.92 g dissolved in anhydrous CH_2_Cl_2_ (10 ml), and dropwise added over a period of 10 min to a solution of 2-nitro-N-methyl-benzenesulfonamide (10 mmol) 2.16g and EtN(i-Pr)_2_ (3 mmol) in CH_2_Cl_2_ (10 ml) at 273 K. The mixture was stirred at r.t. for 4 h. The organic phase was washed with 2N HCl twice, and dried over anhydrous Na_2_SO_4_. The solvent was removed and the residue was purified by flash chromatography (3:1 Cyclohexane:Dichloromethane) to give 1 as a white solid (3.52 mg, 95 %). Single crystals suitable for X-ray measurements were obtained by recrystallization from ethanol and Dichloromethane at room temperature.

### Refinement

H atoms were positioned geometrically and refined using a riding model, with N—H = 0.88 Å, C—H = 0.95 or 0.98 Å and with *U*_iso_(H) = 1.2 (1.5 for methyl groups) *U*_eq_(C,N).

### Crystal data

**Table:**

C_13_H_13_N_3_O_6_S_2_	*F*(000) = 1536
*M**_r_* = 371.38	*D*_x_ = 1.654 Mg m^−^^3^
Orthorhombic, *P**b**c**a*	Mo *K*α radiation, λ = 0.71073 Å
Hall symbol: -P 2ac 2ab	Cell parameters from 37072 reflections
*a* = 13.844 (3) Å	θ = 6.3–55.1°
*b* = 12.942 (2) Å	µ = 0.40 mm^−^^1^
*c* = 16.645 (3) Å	*T* = 153 K
*V* = 2982.2 (10) Å^3^	Platelet, yellow
*Z* = 8	0.60 × 0.56 × 0.08 mm

### Data collection

**Table:**

Rigaku R-AXIS RAPID IP area-detector diffractometer	2630 independent reflections
Radiation source: Rotating anode	2428 reflections with *I* > 2σ(*I*)
graphite	*R*_int_ = 0.027
ω oscillation scans	θ_max_ = 25.0°, θ_min_ = 3.2°
Absorption correction: multi-scan (ABSCOR; Higashi, 1995)	*h* = −16→16
*T*_min_ = 0.733, *T*_max_ = 0.969	*k* = −15→15
34829 measured reflections	*l* = −19→18

### Refinement

**Table:**

Refinement on *F*^2^	Secondary atom site location: difference Fourier map
Least-squares matrix: full	Hydrogen site location: inferred from neighbouring sites
*R*[*F*^2^ > 2σ(*F*^2^)] = 0.032	H-atom parameters constrained
*wR*(*F*^2^) = 0.087	*w* = 1/[σ^2^(*F*_o_^2^) + (0.0473*P*)^2^ + 2.1978*P*] where *P* = (*F*_o_^2^ + 2*F*_c_^2^)/3
*S* = 1.08	(Δ/σ)_max_ < 0.001
2630 reflections	Δρ_max_ = 0.42 e Å^−^^3^
218 parameters	Δρ_min_ = −0.39 e Å^−^^3^
0 restraints	Extinction correction: SHELXTL (Sheldrick, 2001), Fc^*^=kFc[1+0.001xFc^2^λ^3^/sin(2θ)]^-1/4^
Primary atom site location: structure-invariant direct methods	Extinction coefficient: 0.0018 (3)

### Special details

**Table:**

Geometry. All esds (except the esd in the dihedral angle between two l.s. planes) are estimated using the full covariance matrix. The cell esds are taken into account individually in the estimation of esds in distances, angles and torsion angles; correlations between esds in cell parameters are only used when they are defined by crystal symmetry. An approximate (isotropic) treatment of cell esds is used for estimating esds involving l.s. planes.
Refinement. Refinement of F^2^ against ALL reflections. The weighted R-factor wR and goodness of fit S are based on F^2^, conventional R-factors R are based on F, with F set to zero for negative F^2^. The threshold expression of F^2^ > 2sigma(F^2^) is used only for calculating R-factors(gt) etc. and is not relevant to the choice of reflections for refinement. R-factors based on F^2^ are statistically about twice as large as those based on F, and R- factors based on ALL data will be even larger.

### Fractional atomic coordinates and isotropic or equivalent isotropic displacement parameters (Å^2^)

**Table:**

	*x*	*y*	*z*	*U*_iso_*/*U*_eq_	
S1	0.22770 (3)	0.20009 (3)	0.06984 (2)	0.01845 (14)	
S2	0.43344 (3)	0.22433 (4)	0.04145 (2)	0.01991 (15)	
O1	0.49675 (13)	0.12116 (13)	−0.20069 (10)	0.0487 (5)	
O2	0.40368 (12)	0.08365 (11)	−0.10127 (9)	0.0364 (4)	
O3	0.51339 (9)	0.15580 (11)	0.03241 (8)	0.0281 (3)	
O4	0.43340 (9)	0.29881 (11)	0.10485 (8)	0.0275 (3)	
O5	0.16079 (10)	0.12669 (11)	0.03769 (8)	0.0288 (3)	
O6	0.23132 (9)	0.30186 (10)	0.03747 (8)	0.0243 (3)	
N1	0.44722 (12)	0.14579 (12)	−0.14329 (9)	0.0266 (4)	
N2	0.33660 (11)	0.14908 (11)	0.05243 (9)	0.0214 (3)	
N3	0.14942 (13)	0.03260 (13)	0.19054 (11)	0.0366 (4)	
H3B	0.1590	0.0188	0.1394	0.044*	
H3C	0.1251	−0.0150	0.2224	0.044*	
C1	0.43468 (12)	0.25628 (14)	−0.12717 (11)	0.0211 (4)	
C2	0.43337 (13)	0.31966 (16)	−0.19394 (11)	0.0268 (4)	
H2A	0.4441	0.2917	−0.2460	0.032*	
C3	0.41632 (14)	0.42395 (16)	−0.18432 (12)	0.0301 (5)	
H3D	0.4138	0.4678	−0.2300	0.036*	
C4	0.40295 (14)	0.46490 (15)	−0.10843 (12)	0.0298 (4)	
H4B	0.3924	0.5370	−0.1020	0.036*	
C5	0.40499 (13)	0.40078 (15)	−0.04174 (11)	0.0238 (4)	
H5B	0.3961	0.4295	0.0102	0.029*	
C6	0.41971 (12)	0.29552 (14)	−0.04974 (11)	0.0186 (4)	
C7	0.34860 (16)	0.03595 (15)	0.06305 (13)	0.0347 (5)	
H7A	0.4155	0.0166	0.0511	0.052*	
H7B	0.3049	−0.0006	0.0264	0.052*	
H7C	0.3333	0.0171	0.1186	0.052*	
C8	0.21121 (13)	0.20842 (14)	0.17402 (11)	0.0211 (4)	
C9	0.23455 (13)	0.30465 (15)	0.20835 (12)	0.0255 (4)	
H9A	0.2607	0.3580	0.1758	0.031*	
C10	0.21958 (14)	0.32160 (18)	0.28892 (12)	0.0328 (5)	
H10A	0.2351	0.3864	0.3125	0.039*	
C11	0.18139 (14)	0.24220 (19)	0.33523 (12)	0.0359 (5)	
H11A	0.1707	0.2536	0.3909	0.043*	
C12	0.15875 (14)	0.14838 (18)	0.30301 (12)	0.0340 (5)	
H12A	0.1334	0.0957	0.3367	0.041*	
C13	0.17225 (13)	0.12809 (15)	0.22050 (12)	0.0272 (4)	

### Atomic displacement parameters (Å^2^)

**Table:**

	*U*^11^	*U*^22^	*U*^33^	*U*^12^	*U*^13^	*U*^23^
S1	0.0183 (2)	0.0203 (2)	0.0167 (2)	0.00124 (16)	0.00013 (16)	−0.00004 (16)
S2	0.0185 (2)	0.0241 (3)	0.0171 (2)	0.00214 (16)	−0.00052 (16)	−0.00013 (17)
O1	0.0593 (11)	0.0436 (10)	0.0431 (9)	0.0052 (8)	0.0250 (8)	−0.0135 (7)
O2	0.0501 (9)	0.0231 (7)	0.0360 (8)	−0.0032 (7)	0.0085 (7)	−0.0021 (6)
O3	0.0214 (7)	0.0326 (8)	0.0304 (7)	0.0080 (6)	0.0002 (5)	0.0034 (6)
O4	0.0305 (7)	0.0326 (8)	0.0195 (7)	−0.0013 (6)	−0.0024 (5)	−0.0053 (5)
O5	0.0265 (7)	0.0303 (7)	0.0297 (7)	−0.0043 (6)	−0.0045 (5)	−0.0064 (6)
O6	0.0249 (7)	0.0236 (7)	0.0246 (7)	0.0045 (5)	0.0016 (5)	0.0051 (5)
N1	0.0301 (8)	0.0271 (9)	0.0226 (8)	0.0023 (7)	0.0029 (7)	−0.0057 (7)
N2	0.0223 (8)	0.0175 (8)	0.0243 (7)	0.0030 (6)	0.0058 (6)	0.0015 (6)
N3	0.0400 (10)	0.0259 (9)	0.0438 (10)	0.0013 (8)	0.0158 (8)	0.0048 (8)
C1	0.0195 (9)	0.0215 (9)	0.0222 (9)	−0.0018 (7)	0.0022 (7)	−0.0026 (7)
C2	0.0257 (10)	0.0352 (11)	0.0194 (9)	−0.0058 (8)	0.0032 (7)	0.0002 (8)
C3	0.0301 (10)	0.0315 (11)	0.0287 (10)	−0.0056 (8)	0.0013 (8)	0.0107 (8)
C4	0.0310 (10)	0.0213 (10)	0.0371 (11)	−0.0013 (8)	0.0021 (9)	0.0041 (8)
C5	0.0235 (9)	0.0227 (9)	0.0251 (9)	−0.0004 (7)	0.0024 (7)	−0.0027 (7)
C6	0.0163 (8)	0.0220 (9)	0.0176 (9)	−0.0013 (7)	0.0014 (7)	0.0004 (7)
C7	0.0367 (11)	0.0204 (10)	0.0470 (12)	0.0061 (9)	0.0130 (10)	0.0079 (9)
C8	0.0174 (8)	0.0287 (10)	0.0171 (9)	0.0045 (7)	0.0010 (7)	0.0007 (7)
C9	0.0194 (9)	0.0316 (11)	0.0256 (10)	0.0026 (8)	0.0009 (7)	−0.0022 (8)
C10	0.0245 (10)	0.0453 (12)	0.0285 (10)	0.0031 (9)	−0.0014 (8)	−0.0121 (9)
C11	0.0248 (10)	0.0614 (15)	0.0215 (10)	0.0066 (10)	0.0013 (8)	0.0007 (10)
C12	0.0254 (10)	0.0476 (13)	0.0289 (10)	0.0092 (9)	0.0078 (8)	0.0112 (9)
C13	0.0205 (9)	0.0299 (10)	0.0311 (10)	0.0062 (8)	0.0045 (8)	0.0078 (8)

### Geometric parameters (Å, °)

**Table:**

S1—O6	1.4239 (13)	C3—C4	1.382 (3)
S1—O5	1.4307 (13)	C3—H3D	0.9500
S1—N2	1.6711 (15)	C4—C5	1.386 (3)
S1—C8	1.7523 (18)	C4—H4B	0.9500
S2—O3	1.4263 (13)	C5—C6	1.384 (3)
S2—O4	1.4291 (14)	C5—H5B	0.9500
S2—N2	1.6671 (15)	C7—H7A	0.9800
S2—C6	1.7859 (18)	C7—H7B	0.9800
O1—N1	1.218 (2)	C7—H7C	0.9800
O2—N1	1.224 (2)	C8—C13	1.404 (3)
N1—C1	1.465 (2)	C8—C9	1.408 (3)
N2—C7	1.484 (2)	C9—C10	1.375 (3)
N3—C13	1.370 (3)	C9—H9A	0.9500
N3—H3B	0.8800	C10—C11	1.389 (3)
N3—H3C	0.8800	C10—H10A	0.9500
C1—C2	1.381 (3)	C11—C12	1.364 (3)
C1—C6	1.401 (2)	C11—H11A	0.9500
C2—C3	1.380 (3)	C12—C13	1.411 (3)
C2—H2A	0.9500	C12—H12A	0.9500
			
Cg1···Cg2^i^	3.899 (3)		
			
O6—S1—O5	119.70 (8)	C5—C4—H4B	120.0
O6—S1—N2	105.55 (8)	C6—C5—C4	121.02 (18)
O5—S1—N2	104.89 (8)	C6—C5—H5B	119.5
O6—S1—C8	108.79 (8)	C4—C5—H5B	119.5
O5—S1—C8	109.06 (8)	C5—C6—C1	117.86 (17)
N2—S1—C8	108.27 (8)	C5—C6—S2	116.22 (14)
O3—S2—O4	119.85 (8)	C1—C6—S2	125.40 (14)
O3—S2—N2	105.80 (8)	N2—C7—H7A	109.5
O4—S2—N2	108.22 (8)	N2—C7—H7B	109.5
O3—S2—C6	108.28 (8)	H7A—C7—H7B	109.5
O4—S2—C6	106.24 (8)	N2—C7—H7C	109.5
N2—S2—C6	107.99 (8)	H7A—C7—H7C	109.5
O1—N1—O2	123.58 (17)	H7B—C7—H7C	109.5
O1—N1—C1	117.75 (16)	C13—C8—C9	121.31 (17)
O2—N1—C1	118.56 (15)	C13—C8—S1	123.36 (15)
C7—N2—S2	119.96 (13)	C9—C8—S1	115.23 (14)
C7—N2—S1	118.04 (12)	C10—C9—C8	120.18 (19)
S2—N2—S1	120.91 (9)	C10—C9—H9A	119.9
C13—N3—H3B	120.0	C8—C9—H9A	119.9
C13—N3—H3C	120.0	C9—C10—C11	118.7 (2)
H3B—N3—H3C	120.0	C9—C10—H10A	120.6
C2—C1—C6	121.53 (18)	C11—C10—H10A	120.6
C2—C1—N1	115.70 (16)	C12—C11—C10	121.86 (19)
C6—C1—N1	122.68 (16)	C12—C11—H11A	119.1
C3—C2—C1	119.33 (18)	C10—C11—H11A	119.1
C3—C2—H2A	120.3	C11—C12—C13	121.21 (19)
C1—C2—H2A	120.3	C11—C12—H12A	119.4
C2—C3—C4	120.27 (18)	C13—C12—H12A	119.4
C2—C3—H3D	119.9	N3—C13—C8	123.80 (18)
C4—C3—H3D	119.9	N3—C13—C12	119.46 (18)
C3—C4—C5	119.96 (19)	C8—C13—C12	116.72 (19)
C3—C4—H4B	120.0		
			
O3—S2—N2—C7	8.83 (17)	C2—C1—C6—S2	−172.37 (14)
O4—S2—N2—C7	−120.79 (15)	N1—C1—C6—S2	11.1 (2)
C6—S2—N2—C7	124.60 (15)	O3—S2—C6—C5	−134.60 (14)
O3—S2—N2—S1	176.65 (10)	O4—S2—C6—C5	−4.64 (16)
O4—S2—N2—S1	47.02 (12)	N2—S2—C6—C5	111.28 (15)
C6—S2—N2—S1	−67.59 (12)	O3—S2—C6—C1	36.88 (18)
O6—S1—N2—C7	−166.74 (14)	O4—S2—C6—C1	166.83 (15)
O5—S1—N2—C7	−39.44 (16)	N2—S2—C6—C1	−77.25 (16)
C8—S1—N2—C7	76.90 (16)	O6—S1—C8—C13	157.41 (15)
O6—S1—N2—S2	25.21 (12)	O5—S1—C8—C13	25.25 (18)
O5—S1—N2—S2	152.52 (10)	N2—S1—C8—C13	−88.35 (16)
C8—S1—N2—S2	−91.14 (11)	O6—S1—C8—C9	−18.95 (16)
O1—N1—C1—C2	32.8 (2)	O5—S1—C8—C9	−151.11 (13)
O2—N1—C1—C2	−143.45 (18)	N2—S1—C8—C9	95.29 (14)
O1—N1—C1—C6	−150.48 (18)	C13—C8—C9—C10	0.1 (3)
O2—N1—C1—C6	33.2 (3)	S1—C8—C9—C10	176.58 (15)
C6—C1—C2—C3	−0.4 (3)	C8—C9—C10—C11	0.1 (3)
N1—C1—C2—C3	176.31 (17)	C9—C10—C11—C12	0.2 (3)
C1—C2—C3—C4	1.5 (3)	C10—C11—C12—C13	−0.7 (3)
C2—C3—C4—C5	−1.1 (3)	C9—C8—C13—N3	−179.11 (17)
C3—C4—C5—C6	−0.4 (3)	S1—C8—C13—N3	4.8 (3)
C4—C5—C6—C1	1.4 (3)	C9—C8—C13—C12	−0.6 (3)
C4—C5—C6—S2	173.57 (15)	S1—C8—C13—C12	−176.77 (14)
C2—C1—C6—C5	−1.0 (3)	C11—C12—C13—N3	179.44 (19)
N1—C1—C6—C5	−177.53 (16)	C11—C12—C13—C8	0.9 (3)

Symmetry codes: (i) −*x*−1/2, *y*−1/2, *z*−1.

### Hydrogen-bond geometry (Å, °)

**Table:**

*D*—H···*A*	*D*—H	H···*A*	*D*···*A*	*D*—H···*A*
N3—H3C···O1^ii^	0.88	2.52	3.366 (2)	160.
C12—H12A···O2^ii^	0.95	2.59	3.508 (3)	162.
C9—H9A···N3^iii^	0.95	2.59	3.372 (3)	140.

Symmetry codes: (ii) −*x*+1/2, −*y*, *z*+1/2; (iii) −*x*+1/2, *y*+1/2, *z*.
